# A Preclinical Physiological Assay to Test Modulation of Knee Joint Pain in the Spinal Cord: Effects of Oxycodone and Naproxen

**DOI:** 10.1371/journal.pone.0106108

**Published:** 2014-08-26

**Authors:** Jason A. Miranda, Phil Stanley, Katrina Gore, Jamie Turner, Rebecca Dias, Huw Rees

**Affiliations:** 1 Neusentis Research Unit, Pfizer, Cambridge, United Kingdom; 2 Research Statistics, Clinical Research, Pfizer, Cambridge, United Kingdom; University of Texas at Dallas, United States of America

## Abstract

Sensory processing in the spinal cord during disease states can reveal mechanisms for novel treatments, yet very little is known about pain processing at this level in the most commonly used animal models of articular pain. Here we report a test of the prediction that two clinically effective compounds, naproxen (an NSAID) and oxycodone (an opiate), are efficacious in reducing the response of spinal dorsal horn neurons to noxious knee joint rotation in the monosodium iodoacetate (MIA) sensitized rat. The overall objective for these experiments was to develop a high quality *in vivo* electrophysiology assay to confidently test novel compounds for efficacy against pain. Given the recent calls for improved preclinical experimental quality we also developed and implemented an Assay Capability Tool to determine the quality of our assay and ensure the quality of our results. Spinal dorsal horn neurons receiving input from the hind limb knee joint were recorded in anesthetized rats 14 days after they were sensitized with 1 mg of MIA. Intravenous administered oxycodone and naproxen were each tested separately for their effects on phasic, tonic, ongoing and afterdischarge action potential counts in response to innocuous and noxious knee joint rotation. Oxycodone reduced tonic spike counts more than the other measures, doing so by up to 85%. Tonic counts were therefore designated the primary endpoint when testing naproxen which reduced counts by up to 81%. Both reductions occurred at doses consistent with clinically effective doses for osteoarthritis. These results demonstrate that clinically effective doses of standard treatments for osteoarthritis reduce pain processing measured at the level of the spinal cord for two different mechanisms. The Assay Capability Tool helped to guide experimental design leading to a high quality and robust preclinical assay to use in discovering novel treatments for pain.

## Introduction

### Knee joint pain processing

Highly characterized animal models of pain sensitization are employed to study pain mechanisms and aid the discovery of novel clinical treatments. Although the relevance of studying mechanisms of human disease in rodents is in question [1], [2], many of the fundamental mechanisms underlying pain processing are consistent across vertebrates [3], [4]. This is particularly true for sensitized articular tissue and several models attempt to recapitulate symptoms of osteoarthritis including pain [5]–[7]. In particular, 30–40% of knee joint afferents in the rat and cat contain important modulators of pain and hyperalgesia, such as calcitonin gene-related peptide and substance P [8]–[10]. Joint afferents also have a higher proportion of high threshold and ‘silent’ C-fiber afferents [11], which can develop increased mechanical sensitivity when sensitized. Moreover, sensitization is required for many analgesics to be effective against noxious stimulation [12]–[14] suggesting that these abundant afferents are important in pain processing. Together, this suggests that models of articular sensitization may inform not only drug discovery for joint pain but also general pain mechanisms.

The rat monosodium iodoacetate (MIA) model of knee joint sensitization is one of the most extensively characterized in terms of pain behavior and peripheral physiology. MIA intra-articular injection results in large reductions in weight bearing on the sensitized limb and spontaneous mobility that persist beyond 14 days [15]–[17]. Consistent with these behavioral changes, C-fiber primary afferents show increased sustained action potential firing in response to noxious joint rotation and compression as well as increased spontaneous firing [12], [18]. These patterns of response are common to other knee joint sensitization methods [19], [20] as is sustained spiking activity after noxious stimulation has ended [21]–[23] which is hypothesized to be due to sustained C-fiber activity. MIA sensitized neural responses are also susceptible to pharmacological modulation [12]–[14].

Despite the characterization of knee joint pain processing in the periphery of sensitized rats, very little is known about the state of processing in the spinal dorsal horn. Cellular and molecular changes do occur in the spinal cord such as increases in COX-1/-2 [24], proinflammatory cytokines [25], pain related neuropeptides [26], [27], activation of mitogen activated protein kinases [28] and microglial activation [29]–[31], suggesting that pain processing in the spinal cord is important to the behavioral effects in this model. Wide dynamic range neurons receiving direct input from the MIA sensitized knee joint have shown increased spontaneous spiking activity [13] but the majority of spinal cord physiology studies focus on secondary sensitization arising in the ipsilateral paw [32], [33]. Here we report a test of the prediction that two clinically effective compounds, naproxen (an NSAID) and oxycodone (an opiate), are efficacious in reducing the response of spinal dorsal horn neurons to noxious knee joint rotation in the MIA sensitized rat. The objective for these experiments was to develop a high quality *in vivo* electrophysiology assay to confidently test novel compounds for efficacy against pain.

### Assessing experimental quality and assay capability

Recent publications have established there is a high risk of experimental bias and irreproducibility in the majority of preclinical research areas, including pain [34]–[39]. Recommendations for experimental design and reporting have been proposed by the Collaborative Approach to Meta-analysis and Review of Animal Data in Experimental Studies (CAMARADES) [40], the US National Institute for Neurological Disorders and Stroke [41] and the ARRIVE guidelines from the National Centre for the Replacement, Refinement & Reduction of Animals in Research [42]. Adopting these recommendations may also reduce both animal use and the cost of research projects while at the same time increasing confidence in results [43]. This highlights a clear need for a comprehensive tool to guide experimental design and reporting and, ultimately, standardization across preclinical research. We have designed and implemented a broadly applicable tool, the “Assay Capability Tool” (ACT), to guide the development and assess the quality of our drug discovery assays and exploratory experiments in pain physiology. The ACT complements the ARRIVE reporting guidelines by addressing issues of data quality and reproducibility.

Two issues must be addressed to ensure rigorous and repeatable experiments and/or assays: 1) unbiased experimental design and conduct and 2) adequate reporting. Here we report the results from two separate experiments aimed at addressing these issues. Recent meta-analyses in several research disciplines have highlighted a common failure to use key experimental design components that help guard against biased results [34]–[38], [44], [45] including allocation concealment, random experimental treatment allocation, sample size calculation, blinded outcome assessment and replication. In addition, commonly used methods for statistical interpretation of treatment effects are at high risk of introducing bias [46], [47]. Second, several groups have called for improved reporting standards to improve reproducibility, rigorous assessment of results and efficient use of animals [41], [42], [48], [49]. A broadly applicable tool would need to incorporate both of these aspects. Using the ACT led to the foundation of a robust assay in which to test novel compounds for preclinical efficacy and its broader implementation may help guide the standardization of experimental quality across preclinical research.

## Methods

### Experimental animals

Adult male Sprague Dawley rats were ordered from Charles River (Margate, UK), housed in groups of four and given five days to acclimate to the housing facility. Environmental conditions were a temperature of 21°C ±2°, humidity of 55% ±10%, lighting of 350 lux (at bench level) and a 12∶12 light:dark cycle with lights on at 0700 and off at 1900. Animals were housed in 595×380×200 mm cages (Techniplast UK, 1354G Eurostandard Type IV) and given access to rat maintenance food (#801002 RMI [E] Diet, Special Diet Services, Witham, UK) and water *ad libitum*. Environmental enrichment included bedding (LBS, Litaspen Premium B6 grade), one red tinted guinea pig hut (Bio-Serv, cat# K3261), one 10 mm×10 mm×50 mm aspen chew block (LBS, cat# 011590) and one handful of paper wool nesting material (LBS, cat# 033801). During housing, animals were monitored twice daily for health status. No adverse events were observed. At the start of the experiments animals weighed (mean±SD) 202±14 grams. Both the oxycodone and naproxen studies were each conducted using 23 animals with one cell recorded per animal (n = 12 saline versus n = 11 oxycodone and n = 11 saline versus n = 12 naproxen). One animal from each experiment was excluded due to poor electrophysiological isolation of the focal neuron during the recording. The experimenters were blinded to the pharmacological treatment while processing data and making exclusion decisions. All procedures were carried out under an approved Home Office project license (number PPL 80/2578) and in accordance with the Animals (Scientific Procedures) Act, 1986 (UK) (amended 2013). All sections of this report adhere to the ARRIVE Guidelines for reporting animal research [42]. A completed ARRIVE guidelines checklist is included in Checklist S1.

### Animal preparation

**Figure 1:**
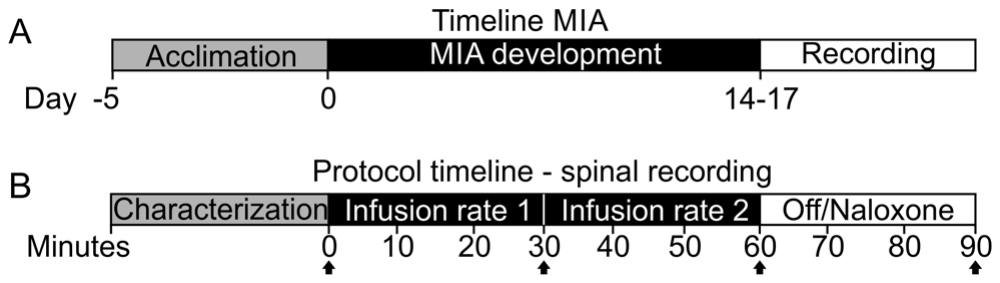
Experimental timelines. (A) MIA Sensitization timeline with recording on a single day between 14–17 days post injection (B) Protocol on the day of recording. Black arrows denote time points at which dried blood spot samples were taken for drug exposure measurement.

Rats were anesthetized with a 3% isoflurane O_2_ mixture and given a single intra-articular injection of monosodium iodoacetate (MIA, Batch # 021M5300V, Sigma-Aldrich Company Ltd., Dorset, UK) through the infrapatellar ligament of the left knee. The MIA was dissolved in 0.9% sterile saline at 0.04 mg/µl, made up immediately before injection and administered in a volume of 25 µl (1 mg per knee). Animals were returned to the home cage until the day of electrophysiological recording, which was 14–17 days post MIA injection (Figure 1A). Previously this protocol has been shown to sensitize knee joint afferents and induce pain behavior [16], [17], [50], [51].

After 14–17 days rats were anesthetized with isoflurane (induction 5% in O_2_ at 4 L/min; reduced to 2.5–3% in O_2_ for surgery at a flow rate of 1 L/min). Cannulae (Portex fine bore polyethylene tubing, 0.5 mm ID, 1 mm OD), were inserted into the right jugular vein and carotid artery. The jugular vein cannula was used for administration of compounds and the carotid cannula allowed both recording of blood pressure and blood sampling for PK analysis. The carotid cannula was pre filled with heparinized saline (250 units/ml) to prevent clotting. Body temperature was maintained throughout the surgery and recording session at 37°C using a homeothermic blanket system (#507221F, Harvard Apparatus Ltd., Kent, UK).

To expose the dorsal spinal cord, an incision was made in the skin on the back of the animal midway between the shoulders and pelvis and the muscle attachments to the dorsal and lateral spinal process cut. The bone was then firmly clamped with lateral bars placed between the dorsolateral and ventrolateral spinal processes at the level of the lumbar enlargement to stabilize the cord for recording. The L3-L5 lumbar cord was exposed by laminectomy, the skin was raised and secured and the cavity filled with warm (37°C) mineral oil (#330779, Sigma-Aldrich Co. Ltd, Dorset, UK) to prevent the exposed tissue from drying out, after which the dura mater was removed.

To stabilize the knee joint for rotation, an incision was made in the skin over the femur of the MIA injected knee and the muscle separated from the femur by blunt dissection. The femur was then clamped to isolate movement to the knee joint during rotation. Once the femur was secured, the isoflurane anesthesia was reduced and maintained at 1.5–2% in O_2_ at a flow rate of 1 L/min during the recording session.

### Electrophysiology recording

Single-unit activity of dorsal horn neurons responding to outward rotation of the knee joint was recorded using fine tungsten microelectrodes (2–5 MΩ, #573200 and #575300, A-M Systems, Sequim, WA). Signals were amplified (head stage NL100, A.C. Preamp NL104, Digitimer Ltd, Welwyn Garden City, UK), low pass filtered at 5 kHz (Neurolog NL125/126, Digitimer), passed through a 50/60 Hz noise eliminator (Humbug, Quest Scientific Instruments Inc., Vancouver, BC), digitized at 20 kHz sampling frequency (CED1401, Cambridge Electronic Designs, Cambridge, UK) and stored on a computer for offline analysis. All of the cells that responded to joint rotation could be activated by firm pressure applied to the joint with a pair of forceps, and this was used as a search stimulus to identify appropriate cells and check that activity was generated primarily from the knee and not the ankle joint. Once an acceptable cell was identified, the left foot was secured in a custom-made protractor device with tissue glue and Micropore Medical tape. Responses were characterized to clockwise rotation of the protractor (outward rotation), which requires more torque per degree, allowing for noxious stimulation at lower angles when compared to inward rotation [52].

All cells that showed some degree of tonic firing on knee rotation and where the knee could be rotated up to 40° beyond the tonic firing threshold were included in the experiment. In any one electrophysiological recording the first cell that responded in this manner was chosen for study irrespective of any other considerations, i.e. presence or absence of baseline activity or any afterdischarge following rotation. After starting the recording, cells were left for two minutes to settle, the foot and knee were then rotated at 20° increasing increments for eight seconds each to establish the tonic spiking activity threshold and therefore defined the range of the rotation stimulus set that extended up to 40° beyond the threshold. Tonic spiking threshold was determined as the lowest rotation angle that elicited at least five spikes occurring during the first six seconds of the rotation after subtracting phasic spiking occurring during the first one second. Ten minutes later, a rotation set was completed in 20° increments up to 40° beyond the tonic spiking threshold to establish the baseline (0 minute time point). Drug or vehicle infusion was then started and rotation sets completed at 10 minute intervals for a further 90 minutes.

### Study conduct

The assay development and study conduct were guided by a novel Assay Capability Tool (ACT). The tool consists of 13 questions and a summary assessment of the experimental capability. Table 1 provides the questions along with explanations of the necessity for each component. The questions are grouped into three sets aimed to address: 1) the alignment of assay capability to the research objectives, 2) managing variation and 3) ensuring objectivity in the conduct of the study. The ACT was used to influence the methodological development of the assay and Table 2 provides the assessment of the experiments in this manuscript.

**Table 1:** Assay Capability Tool.

Question to consider:	Why it is important:		
**Aligning Assay Capability with Project Objectives**			
Q1: Are the scientific objectives forrunning the assay recorded in aprotocol/SOP?	The scientific questions to be answered, the measurements to be obtained and analyzed along with their required precision (as defined by, for example, a standard error or confidence limits) must be stated in the protocol/SOP to prevent data dredging and misinterpretation of the results.		
Q2: What will a successful assayoutcome look like in order to guidedecision making?	Pre-specifying decision criteria leads to clear decisions and ensures unbiased interpretation of results. A target value should be stated for the primary endpoint. Since all assay results include inherent uncertainty, pre-defining what a successful outcome will look like requires a pre-specified level of uncertainty that can be tolerated for acceptable decision making.		
Q3: Is the experimental design, asdescribed in the protocol/SOP, alignedclosely with the objectives?	Is the experimental design and conduct capable of achieving results that meet the objectives? The design and conduct should be revisited in light of current/changing objectives. Once the objectives and definitions of success are defined, consultation with a statistician is essential to ensure the assay is appropriate.		
**Enabling Assay Capability by Managing Variation**			
Q4: Are the assay’s development andvalidation fully documented?	What work has been done in order to verify that an assay is fit for purpose? The answer should identify key lessons/issues/concerns arising from experiments done while the assay was being developed. Assay developers should document validation runs using positive and negative controls and tool compounds to provide benchmarks and reassurance to the users of the resulting data.		
Q5: Have the sources of variabilitypresent in the assay been explored?	All assays exhibit variability and it is important to know what the sources of variability are and their relative sizes. The major sources of variation and the statistical methods that will be used for their control should be summarized in the assay protocol. Understanding and controlling sources of variability in an assay are critical to achieving required precision as captured in the standard errors and confidence intervals for key endpoints.		
Q6: Is the proposed sample size/levelof replication fit for purpose?	An assay that enables a clear decision requires sufficient, but not excessive, precision. Sample size should always be based on what is known about the assay’s variability in the laboratory where it will be run and the quantitative definition of what a successful assay outcome will look like. Relying on historical precedent or published values should not be the default strategy.		
Q7: Is there a comprehensive protocol/SOP detailing study objectives, keyendpoints, experimental design, methodsof analysis and a timetable of activities?	A comprehensive assay protocol supports efficient decisions by specifying the methods to be used to control variation (e.g. randomization, blocking, use of covariates and blinding). It helps to ensure uniformity in assay execution resulting in assay results that are reproducible and comparable from one run to another. It promotes transparency by documenting the actual conditions of the assay. This helps decision makers interpret the results.		
Q8: How is assay performance monitoredover time? What is the plan for reactingto signs of instability?	Repeated assay use should be tracked to detect changing conditions that may affect the interpretation of results and to understand the natural variability of the assay. Quality control (QC) charts are available to monitor the assay consistency of controls, standard or tool compounds over time. Ongoing monitoring is necessary to understand any changes and their implications for interpreting the results and to trigger remediation when necessary.		
**Objectivity in Assay Conduct**			
Q9: Are inclusion/exclusion criteria for theassay specified in the protocol/SOP?	Criteria for the inclusion/exclusion of animals, cells, etc. in an assay should be pre-defined and clearly stated in the protocol/SOP. This ensures all the appropriate data are collected and eliminates selection bias.		
Q10: Is the management of subjectivity indata collection and reporting definedin the protocol/SOP?	There is a need to ensure, through the use of randomization and blinding, that the scientist remains unaware of the treatment that the animal has received. Even when the assay measurement is obtained automatically without human intervention there is possibility for bias, and randomization is essential and blinding is highly recommended. Studies of a long duration should be done in blocks to ensure that no bias is introduced by changing conditions over time.		
Q11: If the raw data are processed (e.g. bysummarization or normalization) prior toanalysis, is the method for doing thisspecified in the study protocol/SOP?	Methods of processing raw data prior to statistical analysis should be clearly stated in the assay protocol/SOP. For example, is it the raw response data, change from baseline or log transformed data that are to be analyzed; or are the raw data summarized into an area under the curve or average? This ensures that assay methods and results can be reproduced and validated.		
Q12: Are rules for treating data as outliersin the analysis specified in theprotocol/SOP?	Rules for treating data as outliers should be clearly stated in the assay protocol/SOP. Rules should be in place for the removal of individual data points, whole animals and dose groups as required. This ensures all the appropriate data are analyzed and eliminates selection bias.		
Q13: Is the analysis specified in thestudy protocol/SOP? Is it fit forpurpose?	The statistical analysis must reflect the study design and assay objectives. Inappropriate analyses can result in misleading conclusions and a false sense of precision. The analysis must incorporate the structure of the data and consultation with a statistician is essential to model the experimental structure appropriately.		
**Summary**			
	**Aligning Assay Capability** **with Project Objectives**	**Enabling Assay Capability by** **Managing Variation**	**Objectivity in Assay Conduct**
Confidence for decision making	Low/Medium/High	Low/Medium/High	Low/Medium/High
Assay Capability Tool Summary	Summarize the evidence for an overall confidence rating in this assay for a given experiment		
**Technical specification**			
Target value	Required effect size for decision making		
Required precision	Depends on the identified acceptable level of risk for a given project		
Required replication	Based on a sample size calculation using data from previous experiments or a pilot study		

**Table 2:** Assay Capability Tool applied to the joint rotation assay.

Question:	Response		
**Aligning Assay Capability with Project Objectives**			
Q1: Are the scientific objectives for running theassay recorded in a protocol/SOP?	See the Introduction section		
Q2: What will a successful assay outcome look likein order to guide decision making?	For the naproxen experiment, an effect size comparable to oxycodone was set as a criteria for success		
Q3: Is the experimental design, as described in theprotocol/SOP, aligned closely with the objectives?	Experiment one, testing oxycodone, was designed as an exploratory test to determine what endpoints are appropriate and what effect size should be expected. Experiment two used the selected primary endpoint to test a different mechanism against the oxycodone benchmark.		
**Enabling Assay Capability by Managing Variation**			
Q4: Are the assay’s development and validationfully documented?	Oxycodone effects are reported for exploratory endpoints along with rationale for the primary endpoint selection		
Q5: Have the sources of variability present in theassay been explored?	Variability in baseline responses is high across cells and potentially high across experimental groups.		
Q6: Is the proposed sample size/level of replicationfit for purpose?	Sample size calculation for naproxen is based on an effect size comparable to oxycodone effects		
Q7: Is there a comprehensive protocol/SOP detailingstudy objectives, key endpoints, experimental design,methods of analysis and timetable of activities?	See the Methods section		
Q8: How is assay performance monitored over time?What is the plan for reacting to signs of instability?	Where this experimental design is used to test novel compounds, oxycodone or naproxen effects will be monitored as positive controls to track assay stability. First response will be to investigate known sources of variability including equipment calibration and inter-experimenter reliability.		
**Objectivity in Assay Conduct**			
Q9: Are inclusion/exclusion criteria for the assayspecified in the protocol/SOP?	Cell inclusion/exclusion is documented in the methods section and is limited to recording quality, animal health and whether they respond to joint rotation within out testable parameters.		
Q10: Is the management of subjectivity in data collectionand reporting defined in the protocol/SOP?	Random allocation to experimental groups, allocation concealment and blinded outcome assessment were implemented and documented as per Sena et al. 2007.		
Q11: If the raw data are processed (e.g. by summarizationor normalization) prior to analysis, is the methodfor doing this specified in the study protocol/SOP?	Change from baseline calculations are performed using ANCOVA and adjusted means are reported. This is documented in the methods section.		
Q12: Are rules for treating data as outliers in theanalysis specified in the protocol/SOP?	Outliers are identified as part of the statistical model fitting process and documented.		
Q13: Is the analysis specified in the study <@?show=[sr]?>protocol/SOP?Is it fit for purpose?	A detailed analysis protocol is included in the methods section		
**Summary**			
	**Aligning Assay Capability** **with Project Objectives**	**Enabling Assay Capability by** **Managing Variation**	**Objectivity in Assay Conduct**
Confidence for decision making	High	High	High
Assay Capability Tool Summary	These experiments are fit for purpose and likely to produce repeatable and reliable data upon which to base further experiments or make preclinical drug research decisions.		
**Technical specification**			
Target value	Both oxycodone and naproxen showed a maximum ratio of approximately 0.2 (80% reduction) for the tonic response		
Required precision	95% confidence intervals around the ratio should not overlap with a ratio value of one.		
Required replication	Sample size calculations suggest approximately 19 replications per group. The addition of future experiments will increase the confidence in this estimate.		

Randomization and blinding are essential components of good study conduct. A randomized drug and vehicle administration schedule was created using the standard = RAND() function in Microsoft Excel. In an effort to reduce animal use in the long term, randomization was done in two separate blocks, with half the drug and control animals randomly allocated to the first block and half to the second. At the end of block one, data were processed and unblinded to determine whether the whole experiment was likely to be futile. This is akin to futility assessment in clinical trials except that, instead of predetermining strict futility criteria, we simply looked for a complete lack of evidence of an effect. Since we did not see this in either of these experiments we continued with the second randomized and blinded blocks. Coded vials containing the treatment were prepared by a third person not involved in the experiment to maintain blinding.

### Drug administration protocols

Figure 1B illustrates the timeline for pharmacological treatments. Rats were i.v. infused with 0.3 mg/ml oxycodone hydrochloride (Sigma-Aldrich Co. LTD, cat# O1378-500MG) dissolved in 0.9% saline, at 0.67 ml/kg/hr for 30 minutes followed by 2 ml/kg/hr for 30 minutes. This infusion protocol was chosen to deliver the highest dose that did not cause severe respiratory depression and fell within the range of clinical plasma concentrations [53]. Vehicle control animals received the same infusion rates with 0.9% saline. During the final 30 minutes, animals randomized to the oxycodone group were infused with 10 mg/ml naloxone hydrochloride (Sigma, cat#N7758-250MG) dissolved in 0.9% saline, at 2 ml/kg/hr for 30 minutes. Animals randomized to the vehicle group were administered saline during the final 30 minutes. The experimenter was blinded to both phases of the treatment. This dose was chosen to ensure a level known to antagonize the antinociceptive effects of oxycodone while avoiding the known antinociceptive effects of Naloxone at low doses [54], [55]. Vehicle control animals received the same infusion rates with 0.9% saline.

Naproxen sodium (Sigma, cat# M1275-5G) was administered at 1.5 mg/ml i.v. dissolved in 0.9% saline, at 1 ml/kg/hr for 30 minutes followed by 2 ml/kg/hr for 30 minutes followed by no infusion for 30 minutes. This dose was chosen to produce an unbound plasma concentration consistent with clinical exposures [56]. Vehicle control animals received the same infusion rates with 0.9% saline.

### PK analysis

In both experiments, 30 µl blood spots were taken at 0, 30, 60 and 90 minutes for blood concentration analysis by Unilabs York Bioanalytical Solutions (Sandwich, UK) using mass spectrometry. Working solutions were prepared in 25% acetonitrile and used to spike sufficient calibration samples and QC samples in blank fresh rat blood. Calibration samples were prepared to generate a calibration line from 1–1000 ng/ml and QC samples were prepared at 5, 50 and 500 ng/ml. A 5000 ng/ml QC was additionally run diluted 7.2 fold within the assay batch. Mass spectrometry was performed on an API5000, with oxycodone monitored using m/z 316.2–241.2 DP 90 CE 39 and naproxen monitored using m/z 231.1–185.1 DP 100 CE 22. Samples were injected (20 µl) into an HPLC-MS/MS system with oxycodone resolved on a Zorbax XDB-C18 50×3 mm 1.8U column and naproxen resolved on an Accucore Polar Premium 50×3 mm 2.6 U column.

### Data processing and analysis

As part of the assay development, four endpoints were measured in the oxycodone experiment: the phasic spike count during the first one second of joint rotation, the tonic spike count during the subsequent six seconds of joint rotation, the ongoing spike count occurring during the 120 seconds preceding the first rotation of a stimulus set, and the afterdischarge spike count 120 seconds following the offset of the last rotation in a stimulus set. Since these responses are counts, they are not normally distributed and the natural log (ln(x+1) to overcome counts of zero) was used to stabilize the variances and reduce the asymmetry of the distributions. All analyses were performed on the log transformed counts, fitting a one-way analysis of covariance using the 0 minute rotation set as the covariate. The results are presented as geometric means, ratios of geometric means and 95% confidence interval estimates of the ratios.

Inferences were made from individual analyses of covariance performed at the 30, 60, and 90 minute rotation sets separately using the 0 minute trial as the covariate. These rotation set time points were identified *a priori* as they represent the end of the separate drug infusions and therefore the time of maximum drug exposure. All analyses were carried out using GenStat Version 11 (VSN International Ltd, Hemel Hempstead, UK).

## Results

### Oxycodone experiment

**Figure 2:**
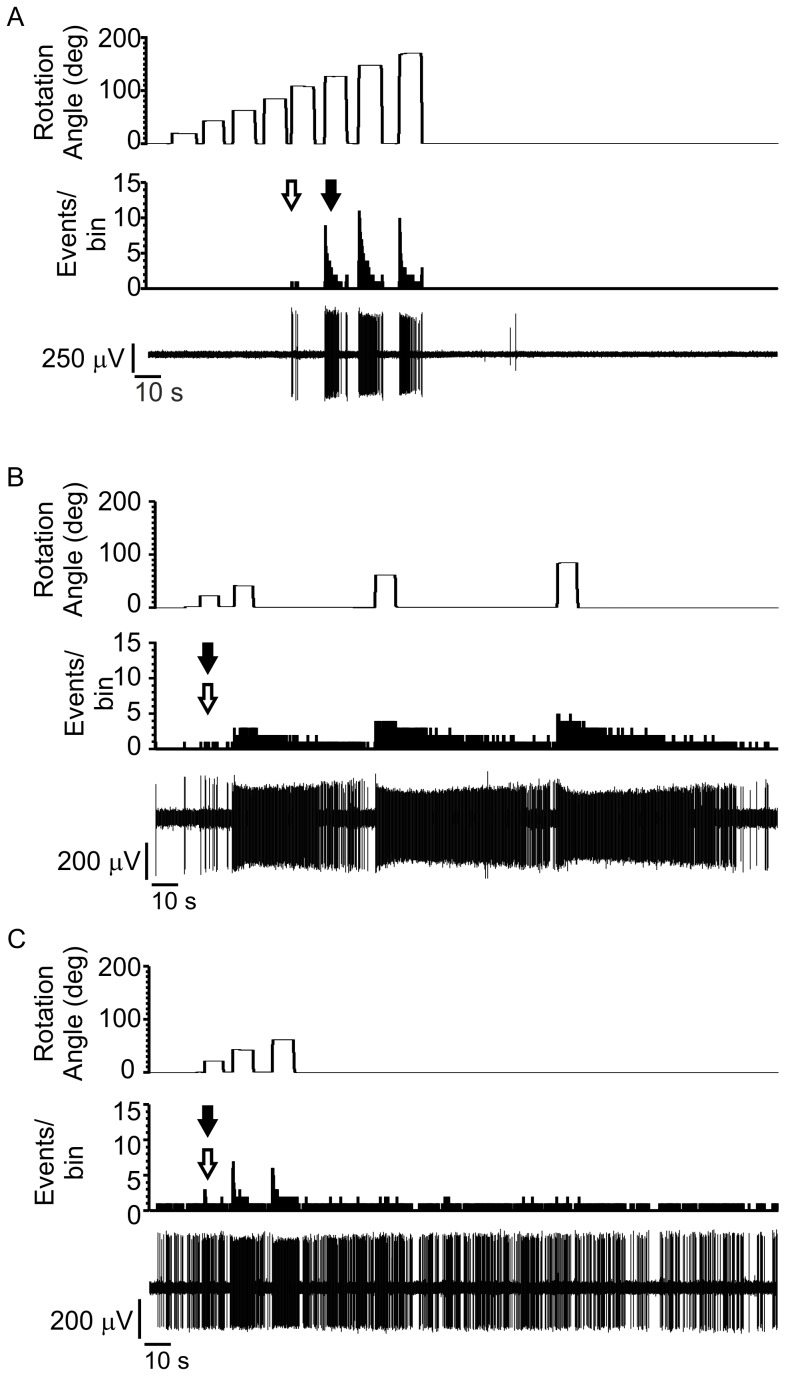
Spinal cord neuron responses to knee joint rotation. For each of (A), (B) and (C) the top trace shows the rotation stimulus set with rotation angle increasing in 20 degree steps and extending at least 40 degrees above the tonic firing threshold. The middle trace shows the PSTH of the response to the rotation set. White arrow denotes phasic threshold, black arrow denotes tonic threshold and bin width = 0.05 ms. The bottom trace shows the neural waveform during the joint rotation stimulus set. (A) Response from a cell showing no ongoing spike activity and high response threshold (B) A cell showing little ongoing spike activity, low threshold and extended firing after stimulus offset (C) A cell showing high ongoing spike activity with a low response threshold.

This was an exploratory experiment with the goal of identifying an appropriate primary endpoint and understanding the interplay between spike reduction and assay variation. Spinal dorsal horn neurons were selected from L3-L4 when they responded predominantly to knee joint rotation. Response types were heterogeneous in their firing patterns. Figure 2 shows three distinct and common response types: high threshold cells with no baseline activity or afterdischarge following the stimulation (Figure 2A), cells with low threshold onset to a rotational force with distinct afterdischarge outlasting the stimulus (Figure 2B), and low threshold cells with ongoing baseline activity (Figure 2C). In general, threshold was not correlated with either the presence or absence of baseline firing or prolonged responses to stimulation, and there were too few cells in this study to determine whether these represented distinct cell types responding to joint rotation.

**Figure 3:**
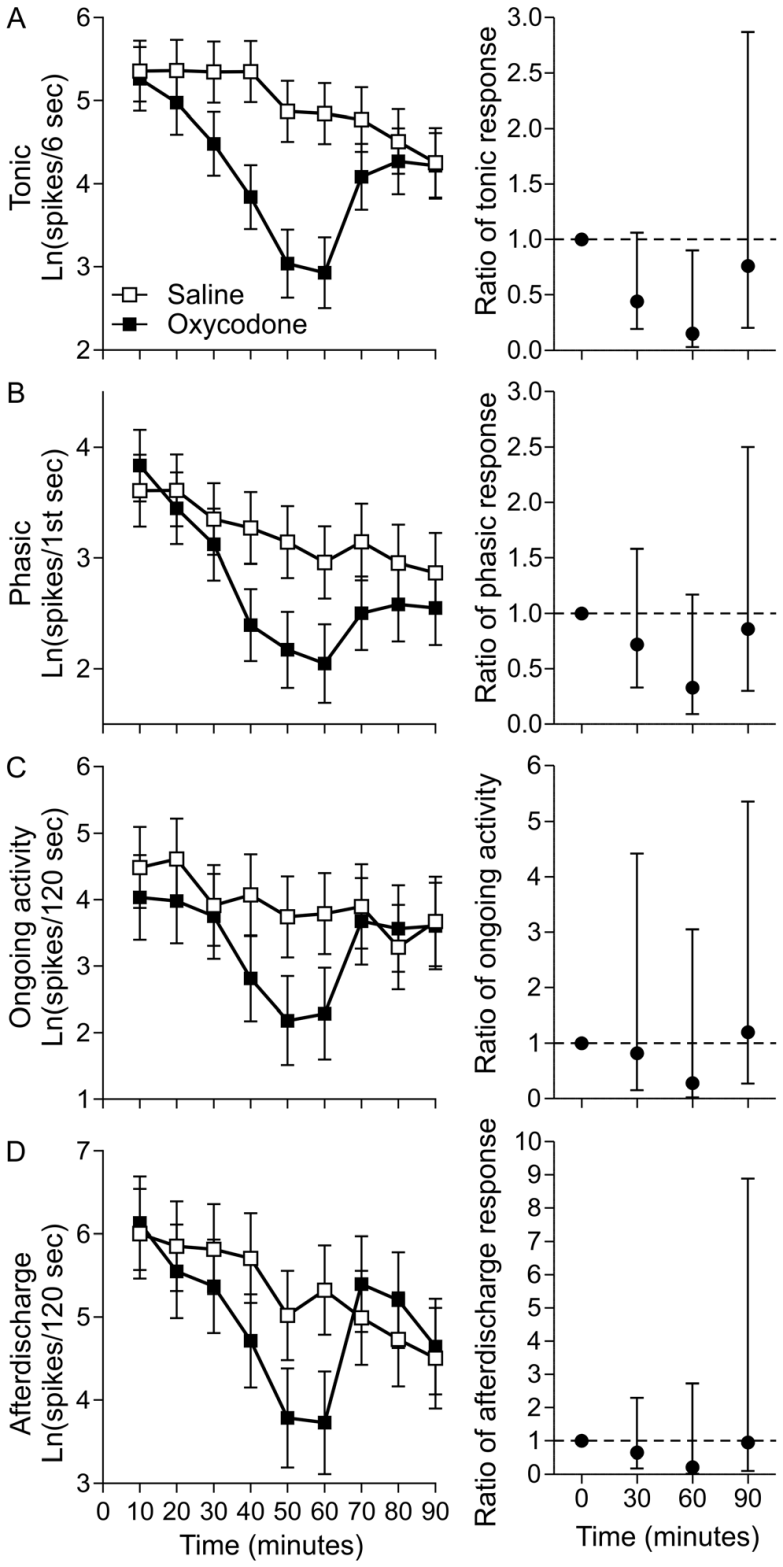
Effects of oxycodone on spinal dorsal horn neuronal activity. Each row shows the results for an exploratory endpoint with ANCOVA adjusted means on the left (error bars ± sem) and the ratios of adjusted means for oxycodone/saline on the right (error bars ±95%CI). Ratio graphs focus on 0, 30, 60 and 90 minutes, the time points at which a following cohort of animals was determined to have (mean±sem) 0±0 nM, 44±6 nM, 217±24 nM and 70±12 nM free plasma concentration of oxycodone respectively. The 90 minute time point is after a 30 minute infusion of 20 mg/kg/hr naloxone with no oxycodone infused. (A) tonic spike count during the six seconds following phasic spiking (B) phasic spiking during the first one second after rotation onset (C) ongoing activity quantified during the 120 seconds preceding each stimulus set (D) afterdischarge during the 120 seconds following the last rotation of each set.

Mean spike count profiles, adjusted for baseline spike count, suggest that oxycodone reduced spike counts in all four endpoints when compared to vehicle controls (Figure 3). Comparing the two groups at the end of each infusion using ratios of geometric means for oxycodone to vehicle and 95% confidence intervals showed that these effects were statistically significant (p<0.05) for tonic responses at the 60 minute rotation set where the unbound plasma concentration of oxycodone reached a mean peak of 217±24 nM (mean±sem) (Figure 3A). The ratio showed an effect size of 0.15 (85% reduction) for tonic spike count. These effects were partially reversed by the addition of naloxone, with statistically non-significant differences between the vehicle and oxycodone/naloxone groups at the end of the naloxone infusion (90 minute time point).

Three of the 11 animals in the oxycodone group showed complete respiratory arrest before the end of the 60 minute trial where plasma levels were highest. In all cases, respiration was recovered by administering naloxone resulting in missing data points for the 60 minute trial. To determine whether the inclusion of these animals in the analysis resulted in a bias toward a larger effect size in earlier trials, we excluded all data from the three animals and reanalyzed the data. The removal of these data points did not change the effect size but the reduction in sample size did reduce statistical power and thus the 95% confidence intervals were wider (data not shown). Since effect size was not impacted for earlier trials, data from these animals were included in the analysis. This study was used to estimate the within cell and between cell variability which was used to estimate the sample size required to detect oxycodone-like effects in future studies.

### Naproxen experiment

**Figure 4:**
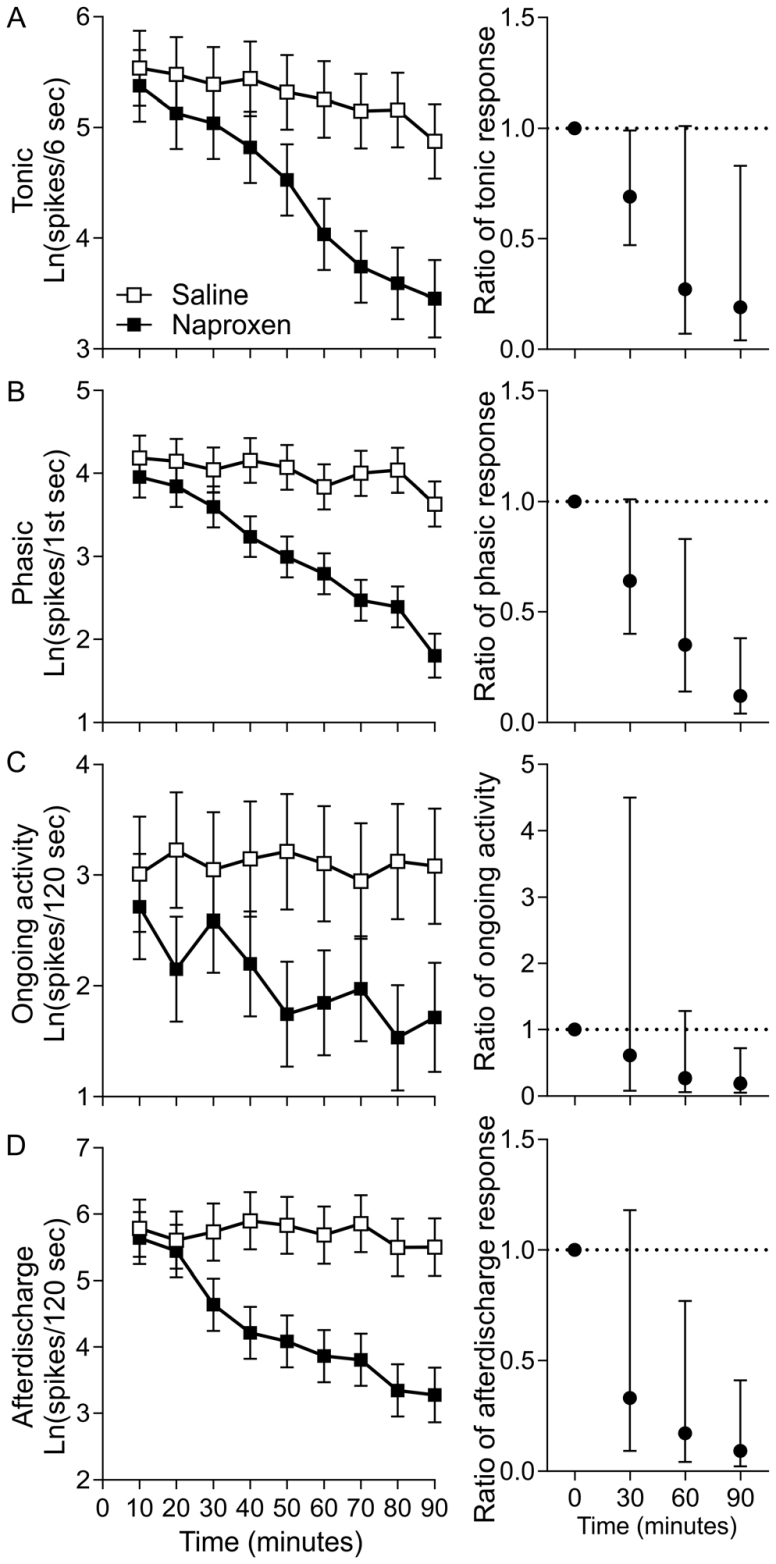
Effects of naproxen on spinal dorsal horn neuronal activity. Each row shows the results for an exploratory endpoint with ANCOVA adjusted means on the left (error bars ± sem) and the ratios of adjusted means for naproxen/saline on the right (error bars ±95%CI). Ratio graphs focus on 0, 30, and 60 minutes, the time points at which these animals were determined to have (mean±sem) 0±0 nM, 165±28 nM and 697±45 nM free plasma concentration of naproxen respectively and 90 minutes which is after 30 minutes of no infusion resulting in 567±34 nM free plasma concentration. (A) tonic spike count during the six seconds following phasic spiking (B) phasic spiking during the first one second after rotation onset (C) ongoing activity quantified during the 120 seconds preceding each stimulus set and (D) afterdischarge during the 120 seconds following the last rotation of each set.

This experiment followed the same methods as the oxycodone study. As with oxycodone, baseline adjusted mean spike count profiles suggest that naproxen reduced spike counts in all four endpoints when compared to vehicle controls (Figure 4), but tonic spike activity during joint rotation was designated as the primary endpoint. This was due to the observation that tonic spiking was associated with increased rotation angle into the noxious range and was the measure most affected by oxycodone treatment. Comparing the two groups at the end of each infusion using ratios of geometric means for naproxen to vehicle and 95% confidence intervals show that these effects were nearly statistically significant for the 60 minute rotation set where unbound plasma concentration of naproxen reached a mean peak of 697±45 nM (Figure 4B). When the i.v. infusion was turned off for 30 minutes, mean plasma concentration reduced to 567±34 nM and the reduced tonic spiking was statistically significant (p<0.05) at the 90 minute rotation set with ratios showing an effect size of 0.19 or an 81% reduction. Unlike for oxycodone, naproxen treatment significantly reduced (p<0.05) spike counts for all three secondary endpoints at the 90 minute rotation set (Figure 4).

## Discussion

The overall objective for these experiments was to develop a high quality *in vivo* electrophysiology assay to confidently test novel compounds for efficacy against pain. Assay development is an evolving process of experimentation and refinement. The experimental methods and results presented here were developed under the guidance of a novel tool, the Assay Capability Tool (ACT), to strive for the highest possible standards of experimental conduct. The ACT was developed to guide both the development of assays and the assessment of their capability to generate reliable data. The tool is particularly useful in standardizing decision making in drug discovery but can also be applied to published experiments. This aids in the estimation of the confidence one can have in the validity of the results and guides follow-up studies.

We started by running a small pilot study with oxycodone to assess whether this model was able to detect electrophysiological changes following drug administration. This pilot study indicated that the experimental approach was viable (data not shown). We then moved on to test novel predictions about the efficacy of oxycodone and naproxen, two clinical standards of care for knee joint pain, which have differing mechanisms of action. Our next experiment was designed to further explore the role of oxycodone versus vehicle-treated controls in modulating sensitized knee joint rotation processing in the spinal cord. This provided the information to design a follow-up study to test the validity of tonic spiking activity as a primary endpoint and to test the role of naproxen.

In the oxycodone experiment, our objective was to test predictions about the role of opioid receptors in modulating four knee joint rotation response types, each with their own putative underlying physiological mechanisms. Although phasic, ongoing, and afterdischarge spiking did show reductions at 0.3 mg/kg oxycodone, the high levels of variation in the effects do not provide evidence for a strong role of opioid receptors in modulating the mechanisms underlying these responses. Tonic spike counts showed a mean reduction of 85% with 0.3 mg/kg oxycodone. Given that tonic spiking profiles in the spinal cord are consistent with the coding of stimulus intensity, these results suggest that opioid receptors play a strong role in modulating this coding, particularly in the noxious range. Surprisingly, no study to our knowledge has tested the effects of oxycodone against a physiological endpoint in the spinal cord, where cognitive confounds are eliminated. Nonetheless, these results are consistent with the clinical effectiveness of oxycodone in joint pain. Oxycodone is likely acting on the central nervous system as opposed to peripheral nerves since µ-opioid receptor numbers are severely reduced in sensitized rat knee joints [57]. Whether this reduction occurs in MIA sensitized joints and which opioid receptor subtypes contribute to the oxycodone effect is not known.

Comparisons between clinical and preclinical effective doses depend on differences between route of administration and potential interactions between drug effects and the anaesthetic. Despite these complications, our effective doses are either consistent with or below those in clinical and preclinical behavioral studies. For oxycodone, similar magnitude reductions have been reported in rat behavioral pain assays that require much higher doses of between 4–10 mg/kg [58]–[60]. Our doses, and resulting plasma concentration levels, are comparable to maximum doses administered to humans [61]–[63] whereas doses in most behavioral assays are much higher and overlap with severe adverse events in rats such as loss of righting reflex and catalepsy [60]. One exception is that for pain assessed by observing spontaneous behavior, such as vertical activity (rearing), other opiates show efficacy at lower doses compared to evoked behavior such as paw withdrawal [51]. This suggests that careful consideration must be made when attempting to make clinically effective dose predictions from preclinical data and that behavioral and physiological data together may better determine the validity of those predictions.

The results of this experiment also provided information to answer important questions raised in the ACT. Often in preclinical research, exploratory work must be done to adequately answer most of the questions in the ACT. At this stage, the ACT guides experimental design to incorporate components that increase confidence in the data generated in the experiments. This starts with a clearly stated scientific objective and associated experimental methods (Table 1; Q1, Q3) as well as methods to manage subjectivity in data collection and reporting, such as allocation concealment, and adhering to the ARRIVE guidelines (Q10). However, it was difficult in the oxycodone experiment to ensure that the experimenter was blind to the treatment where noticeable effects on respiratory rate were evident in some cases and respiratory rate is monitored to assess adequacy of anesthesia.

The oxycodone experiment was also designed to produce data that allowed for the first analysis of effect size for future decision-making (Q2), experimental variability (Q5), sample size calculations (Q6), primary endpoint determination (Q7) and appropriate data analysis methods (Q9, Q11, Q12). More specifically, we had evidence that tonic spiking was an appropriate primary endpoint and that an 85% reduction would resemble an oxycodone-like effect. We also determined that the statistical structure of data sets from this type of experiment require natural log transformation, analysis of covariance (ANCOVA) baseline correction and a simpler statistical model that does not rely on potentially misleading estimations (Q13).

To identify the most appropriate statistical analysis method, an initial exploratory analysis was carried out using a repeated measures linear mixed effects model. This included data from all the rotation sets to explore the shape of the response to drug over time. The mixed model was fitted using residual (or restricted) maximum likelihood (REML) with between cell (one per animal) and between repeated measures within each cell as random effects; and treatment group, time, and the treatment by time interaction as fixed effects. The values at baseline (0 minute rotation set) were included as a covariate in the model to reduce the between cell estimate of variability. A heterogeneous first order autoregressive correlation structure was used to model the within cell correlations allowing for unequal variances across the time points. An unstructured correlation matrix was also tried but the REML failed to converge. The heterogeneous first order autoregressive structure may be reasonable due to the equally spaced time points, but the quality of the fit and the assumptions made could not be tested or checked. The complexity of this model and the potential for misleading estimates of treatment means and standard errors [68], [69] meant that it was unwise to make inferences from the model. ANCOVA was preferred to the less sophisticated, and potentially biased, methods of correcting for baseline such as percent change and subtraction as it uses the data to estimate the appropriate correction to minimize the residual variability [46], [47]. The estimated means from the ANCOVA represent the expected response at a typical (average) baseline response, so all comparisons of means are ‘corrected’ for inequalities at baseline using this method. The separate analyses at the specified time points are preferred because they are less complex and are justified based on the comparisons of interest being specified *a priori*.

The estimated effect size and level of variability were used to estimate the required sample size for future studies. Despite finding a statistically significant reduction with 12 animals per group, the sample size estimation suggested that 19 animals would be required to be confident in detecting the same effect size in a follow up study. Balancing this fact with the desire to reduce the use of animals, we chose to continue with 12 animals per group for the naproxen study. At this point in the development of an assay sample sizing should only be viewed as a guide because the estimates of both the effect size and associated variability are approximate and come with much uncertainty attached to them.

The cell selection criteria for these experiments were intentionally broad with the only requirement being sustained spiking activity in response to knee joint rotation. We made no assumptions that a particular endpoint was more likely to be affected by the pharmacological treatments. This contributed to a high level of heterogeneity in response parameters such as response threshold, presence or absence of ongoing activity and degree of afterdischarge. Heterogeneous response types across cells suggest that different classes of cells exist with different mechanisms underlying their response properties. This leaves open the possibility that compounds acting on some mechanisms may only affect a subsample of cells. This could result in a bimodal distribution in the effectiveness of a given compound across cells that could in turn result in an equivocal outcome. We have yet to see such a pattern in response to either oxycodone or naproxen in subsequent runs of this assay. The sample size chosen for these experiments would not be adequate to confidently reveal effects on only a subset of cells but, if such a pattern emerged, a follow-up study would be required to confirm cell specific effects. Such a follow-up study would likely need a larger sample size or narrower cell selection criteria. As we continue to run these experiments to test novel compounds with naproxen and oxycodone as positive controls and saline as a negative control, we will collate a larger dataset with which to investigate the effects on particular cell responses using larger sample sizes. In future studies this may allow us to set cell selection criteria and appropriate sample sizes to test if novel compounds target specific cell types or mechanisms.

We are also performing an informal interim analysis for futility halfway through the experiment. When using such experiments as assays to discover novel therapies, this approach could result in a large reduction in animal use by stopping large studies that are very unlikely to produce positive evidence to support a hypothesis. This approach is in line with the guidelines of the National Centre for the Replacement, Refinement and Reduction of Animals in Research (London, UK).

Being guided by the ACT and the answers to its questions (as summarized in Table 2) informed the design and interpretation of the follow-up experiment with naproxen.

In the naproxen experiment, our objective was to test the prediction that COX-1/COX-2 inhibition reduces the primary endpoint of tonic spiking activity in response to noxious joint rotation. Tonic spike counts showed the largest mean reduction of 81% at 90 minutes after the start of the low dose infusion. This time point is 30 minutes after reaching the peak dose of 1.5 mg/kg (mean of 697 nM free plasma exposure) suggestive of possible hysteresis in this measurement for this mechanism. As with oxycodone, the results are consistent with the clinical effectiveness of naproxen in joint pain. In humans suffering from mild to moderate osteoarthritis, a similar magnitude in the reduction in pain scores has been reported with 5–15 mg/kg oral naproxen doses [64]. Differences in blood protein binding for naproxen between humans and rats [65], [66], suggests that our i.v. administered dose produces unbound concentrations within the clinical therapeutic range. Furthermore, similar magnitude effects on weight distribution in MIA treated rats can be produced by oral doses between 5–18 mg/kg [17], [50] which also appear to be similar to clinical exposures [67].

Given that different pharmacological mechanisms may affect different physiological responses, it is still of value to collect secondary exploratory measures although in future experiments all positive results would require follow up experiments. For the two experiments presented here, tonic spiking activity was the only endpoint that was modulated by both oxycodone and naproxen. Naproxen also significantly reduced measures of neuronal excitability with a large reduction in afterdischarge and a moderate reduction in ongoing activity. These two measures have been of interest when testing hypotheses about neuropathic pain and central sensitization [68], [69]. Since they show particularly high variation across cells, a larger sample size may be required in order to provide a repeatable result if they are identified as a primary endpoint. In the current study, both measures suggest that naproxen may have an effect of reducing the neuronal excitability seen in sensitized pain conditions that warrants further investigation.

Data generated and knowledge gained from all preceding experiments is used in the ACT to define objectives and refine the experimental design and conduct of future studies. This report constitutes the documentation of the experimental design as a foundation for future use as an assay in drug discovery (Q4). Lastly, by including in future experiments at least one of the two compounds tested here along with tests of vehicle, we can monitor assay stability over time to detect any unanticipated variability (Q8). This will ensure that the data produced from this assay in the future will be reliable and directly comparable with previous data, and decisions can be made in the context of the whole experimental system rather than a single experiment.

We would recommend following a structured ACT to gain greater insight into the capability of an assay to better inform future experiments prior to their commencement, thus leading to decisions being made with higher confidence placed on the data generated.

## Supporting Information

Checklist S1 Completed “The ARRIVE Guidelines Checklist” for reporting animal data in this manuscript.
(PDF)

Dataset S1 Oxycodone dataset for all endpoints.
(CSV)

Dataset S2 Naproxen dataset for all endpoints.
(CSV)
